# Comparative Metagenomics of Toxic Freshwater Cyanobacteria Bloom Communities on Two Continents

**DOI:** 10.1371/journal.pone.0044002

**Published:** 2012-08-29

**Authors:** Morgan M. Steffen, Zhou Li, T. Chad Effler, Loren J. Hauser, Gregory L. Boyer, Steven W. Wilhelm

**Affiliations:** 1 Department of Microbiology, The University of Tennessee, Knoxville, Tennessee, United States of America; 2 Graduate School of Genome Science & Technology, The University of Tennessee, Knoxville, Tennessee, United States of America; 3 Oak Ridge National Laboratory, Oak Ridge, Tennessee, United States of America; 4 Department of Chemistry, College of Environmental Science and Forestry, State University of New York, Syracuse, New York, United States of America; Uppsala University, Sweden

## Abstract

Toxic cyanobacterial blooms have persisted in freshwater systems around the world for centuries and appear to be globally increasing in frequency and severity. Toxins produced by bloom-associated cyanobacteria can have drastic impacts on the ecosystem and surrounding communities, and bloom biomass can disrupt aquatic food webs and act as a driver for hypoxia. Little is currently known regarding the genomic content of the *Microcystis* strains that form blooms or the companion heterotrophic community associated with bloom events. To address these issues, we examined the bloom-associated microbial communities in single samples from Lake Erie (North America), Lake Tai (Taihu, China), and Grand Lakes St. Marys (OH, USA) using comparative metagenomics. Together the *Cyanobacteria* and *Proteobacteria* comprised >90% of each bloom bacterial community sample, although the dominant phylum varied between systems. Relative to the existing *Microcystis aeruginosa* NIES 843 genome, sequences from Lake Erie and Taihu revealed a number of metagenomic islands that were absent in the environmental samples. Moreover, despite variation in the phylogenetic assignments of bloom-associated organisms, the functional potential of bloom members remained relatively constant between systems. This pattern was particularly noticeable in the genomic contribution of nitrogen assimilation genes. In Taihu, the genetic elements associated with the assimilation and metabolism of nitrogen were predominantly associated with *Proteobacteria,* while these functions in the North American lakes were primarily contributed to by the *Cyanobacteria*. Our observations build on an emerging body of metagenomic surveys describing the functional potential of microbial communities as more highly conserved than that of their phylogenetic makeup within natural systems.

## Introduction

The theory that microbial community structure dictates the function of that community has recently been called into question [1], [2]. Previously, laboratory studies using enzyme assays and 16S rRNA gene phylogeny provided evidence for the importance of phylogenetic identity to community function [3], [4]. A shift to whole community shotgun metagenomics has, however, allowed for a more comprehensive examination of microbial functional genes present in a wealth of natural environments. This technique circumvents the need for culture-based analysis and is more representative of natural community structure and functional potential. The trend emerging from this work indicates that the function of microbes within the environment is often more highly conserved than their phylogenetic/taxonomic identity [1], [5].

One environment of particular concern is the freshwater systems that have in recent years been increasingly inundated by toxic cyanobacterial blooms. These blooms have been responsible for the deterioration of freshwater systems with increasing frequency and intensity and are commonly dominated by colonial cyanobacteria of the genus *Microcystis* [6]–[8]. Toxins produced by bloom-associated cyanobacteria have been shown to adversely affect health of animals and humans. The microcystins, cyclic secondary metabolites produced by members of the genera *Anabaena*, *Microcystis*, and *Planktothrix*, are hepatotoxins that have been associated with human liver and colorectal cancers [9]–[11]. Other secondary metabolites produced by *Microcystis* have been linked to phytoestrogenic effects in fish [12]. *Microcystis aeruginosa* is commonly dominant within freshwater blooms and has been shown to impact local ecology by disruption of the food web and induction of hypoxia in large lake systems [13], [14].

**Table 1:** Bloom sample metadata.

	Collection Date	Water temp. (°C)	*Chl* a (mg/L)	Microcystin (µg/gdw)	pH
Erie	08/19/2009	25.8	11.7±0.2	<0.05 g/L	8.61
Taihu	05/30/2009	24.1	14.2±0.3	60.5±10.8	8.11
GLSM	07/19/2010	27.0	10.9	537±117	8.8

*Microcystis*-dominated blooms have been observed in the western basin of Lake Erie annually since the 1990s. This freshwater system is a commonly studied model of toxic bloom development and persistence [6], [15]. Lake Tai (hereafter referred to by its Chinese name *Taihu,* which translates to grand or great (*tai*) lake (*hu*)) is a shallow eutrophic lake that has experienced annual cyanobacterial blooms for the last three decades. The Taihu watershed supports upwards of 40 million people and receives inputs from both industrial and agricultural sources. During the last decade Taihu has experienced *Microcystis* bloom events on an unprecedented scale: in 2007 authorities shut down water intakes, resulting in a freshwater shortage for the 4 million residents of the city of Wuxi [16]. In contrast, Grand Lakes St. Marys (GLSM) is a smaller (54.6 km^2^) inland lake in Ohio that has recently experienced recurring highly toxic cyanobacteria blooms during summer months. While GLSM also experiences high concentrations of microcystin during bloom events, this lake is dominated by the filamentous cyanobacterium *Planktothrix* spp. (RML McKay, personal communication). To this end, GLSM provides a novel and contrasting study site (relative to Taihu and Lake Erie) whereby each lake experiences eutrophic conditions leading to the presence of the toxin microcystin, but in GLSM that toxin is produced by a cyanobacterium with a different physiological ecology. Examination of these contrasting freshwater cyanobacterial blooms models may provide insight on the environmental and genetic factors that influence bloom formation worldwide.

Despite our ability to examine *Microcystis* within bloom communities, questions remain regarding the environmental triggers that facilitate bloom formation and persistence. Previous research has shown that numerous abiotic factors, including nutrient input and air temperature, are important during cyanobacterial bloom formation, [17]–[19] although there is a current schism regarding which nutrient inputs most exacerbate bloom formation [20], [21]. Recent work has demonstrated that concentrations of both nitrogen and phosphorus influence success of freshwater cyanobacteria in eutrophic environments [19], [22]–[24]. Moreover, freshwater cyanobacteria thrive at temperatures above 25°C, and increasing mean temperatures associated with recent global climate change have been associated with the increasing frequency of these toxic bloom events [25]–[27].

**Figure 1:**
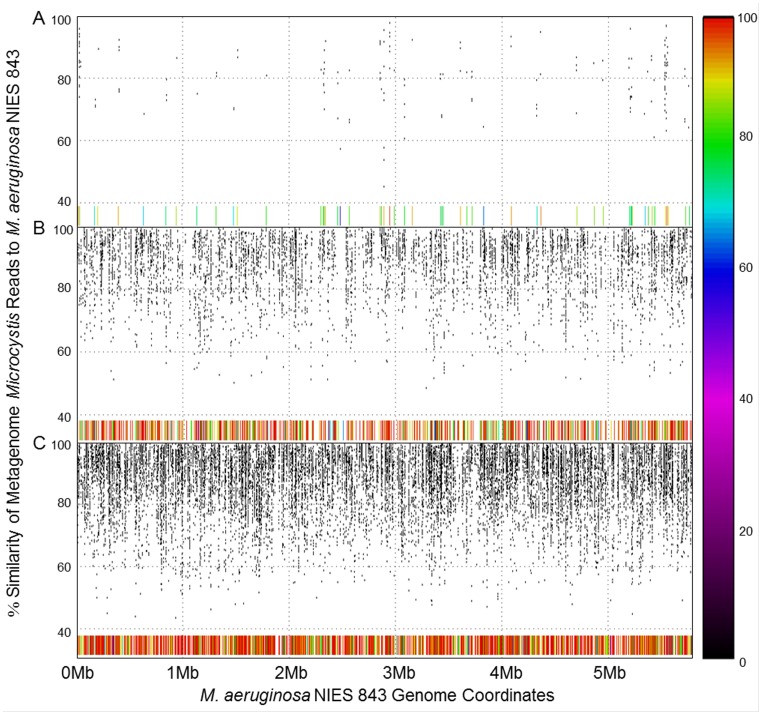
Recruitment plots of metagenomes to Microcystis aeruginosa NIES 843. Recruitment plots of environmental *Microcystis* reads from each metagenome to the *M. aeruginosa* NIES 843 genome. A) Recruitment of GLSM *Microcystis* reads to NIES 843. B) Recruitment of Taihu *Microcystis* reads to NIES 843. C) Recruitment of Erie *Microcystis* reads to NIES 843. Position along the x-axis indicates position along the genome of *M. aeruginosa* NIES 843 from zero to 5.8 Mbp and position along the y-axis indicates percent similarity of recruited sequences. The bar along the bottom is a secondary indicator of percent similarity to the reference genome. See Table S2 for locations of potential MI’s within each recruitment.

**Figure 2:**
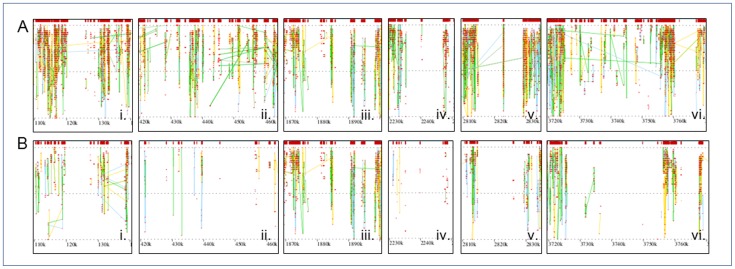
Metagenomic islands (MIs) of bloom-associated *Microcystis*. Metagenomic islands (MIs) identified in the Lake Erie and Taihu *Microcystis* recruitments. Six regions in Taihu and three in Erie qualified as MIs. MIs were defined as regions of ≥10 kb in length that have an average similarity of less than 25% to the reference genome and less than 25% total coverage of the corresponding base pairs of the reference sequence. A) View of MIs of Lake Erie *Microcystis* recruitment. Boxes ii, iii, and vi were found to be MIs in Taihu, but did not satisfy our criteria in Lake Erie. E) View of MI regions in Taihu *Microcystis* recruitment.

Currently, we have little information regarding bloom communities as a whole, in terms of either phylogenetic composition or functional role. Indeed recent work suggests companion heterotrophs may be essential for cyanobacteria biomass growth in response to the addition of certain nutrient sources [28], highlighting the importance of resolving the ecology of this complex community. To gain insight into the role of bloom microbial community members and better understand differences between geographically distinct freshwater microbial communities, we have applied comparative metagenomics to the microbial populations of three toxic freshwater blooms. This approach has enabled us to examine not only the toxin-producing organisms, but also the companion heterotrophic community and their potential role in driving these blooms. In addition, we have used environmental genomic data to identify potential differences in genomic content in bloom-associated *Microcystis* spp. and a model lab isolate (*Microcystis aeruginosa* NIES 843) to further the aim of establishing the core genome of *Microcystis*. This information provides new insight on factors that may be critical to toxic freshwater blooms and which may prove crucial to future mitigation efforts.

## Methods

### Sample Collection

Lake Erie was sampled during an August, 2009 cruise aboard the CCGS *Limnos*. Water was taken from the surface in a 20 µm net tow at Environment Canada station 589 (Erie Harbor, PA; 42° 08′ N 80° 07′ W). Biomass was stored at −80°C until extraction. Taihu was sampled during a large surface bloom in May, 2009 near the Taihu Laboratory for Lake Ecosystem Research (TLLR, 31°27′ N, 120°12′ E) using 20 µm Nytex™. Samples were stored at −20°C until extraction. The GLSM (40°31′ N, 84°24′ W) bloom sample was collected using a 20 µm plankton net during the July 2010 bloom event and stored at −20°C until extraction. The 20 µm size is a commonly used technique in limnology and allowed for collection of *Microcystis,* other large cyanobacteria and the microbial community associated with these cells. Nutrient and toxin measurements at each location were made as previously described [17], [19], [29]. Metadata for each site are listed in Table 1.

### DNA Extraction, Sequencing, Assembly, and Annotation

Genomic DNA was extracted from all samples using the MoBio PowerWater® DNA Isolation Kit (MoBio Laboratories, Inc., CA, USA). The GLSM sample required an additional wash step, using sterile CT medium [30], [31] to remove organic impurities that interfered with nucleic acid purity. The quality and quantity of DNA was measured by spectrophotometric quantification in a NanoDrop 1000 (Thermo Fisher Scientific, Inc., DE, USA) and agarose gel electrophoresis. Extracted DNA was stored at −80°C prior to sequencing.

A total of 500 ng of genomic DNA per sample was used for library preparationand sequenced according using a Roche 454 GS FLX platform with Titanium chemistry (Roche/454-Life Sciences, Branford, CT, USA) at the UT/ORNL Joint Institute of Biological Sciences. We generated a total of 533 Mbp and 1.36 million reads with an average read length of 394 bp (Table S1). The MG-RAST v.3.0 online server quality control pipeline was used to remove reads of poor quality and short length before annotation and analysis of metagenomics data [32]. Pipeline parameters were kept at the default settings. Sequences were dereplicated and filtered by length, removing sequences more than 2.0 standard deviations from the mean sequences length. Sequences with five or more non ATCG characters were removed. Recruitment of all metagenome reads post-QC processing to the *Microcystis aeruginosa* NIES 843 genome as was performed using the SeqMan NGen® (DNASTAR, Inc., WI, USA) software package using manufacturer’s suggested default parameters established for 454 datasets. Reannotation of the NIES 843 reference genome and annotation of the environmental *Microcystis* genes was performed using Prodigal [33].

Metagenome reads were subjected to a BLASTx comparison against the NCBI nonredundant database. Community taxonomy and function were analyzed in the MEGAN v.4.61.6 analysis toolset [34], [35]. To confirm reproducibility of taxonomic assignments, reads were also uploaded to the MG-RAST server for taxonomic and functional potential analysis using a minimum *e-*value cutoff of 1e-5, minimum percent identity of 65% and a minimum alignment length of 50 amino acids. COGs (Clusters of Orthologous Groups) were used to assign function [36]. For MG-RAST comparative analyses, the GenBank database was used for phylogenetic analysis and RefSeq was used to annotate functional genes using the parameters described above [37]. Phylogenetic comparisons were made using percent abundance within each library post quality control analysis. COG and functional gene comparative analyses were performed using reads normalized to the library with the greatest total number of hits to the COG database (Taihu). Sequences generated in this study are available on the MG-RAST online server (http://metagenomics.anl.gov/) under the identification numbers 4467029.3 (Erie), 4467058.3 (Taihu), and 4467059.3 (GLSM).

### *Microcystis* Genome Recruitment

Metagenomes were annotated using the RefSeq database (MG-RAST) with an *e-*value minimum of 1e−5, a 65% identity cutoff, and a minimum alignment length of 50. All reads annotated as *Microcystis* from each metagenome were recruited to the genome of *Microcystis aeruginosa* NIES 843 using MUMmer 3.22 [38]. The PROmer algorithm was used for alignment. Potential metagenomic islands were initially identified manually in the Lake Erie and Taihu recruitment plots as regions of 10 kb or longer with less than average recruitment as established in Rodriguez-Valera et al [39]. These regions were defined as MIs if they had less than 25% average similarity with the reference and coverage of the reference in the region was less than 25%. Genetic elements associated with these regions not present in the metagenomes were identified manually.

**Figure 3:**
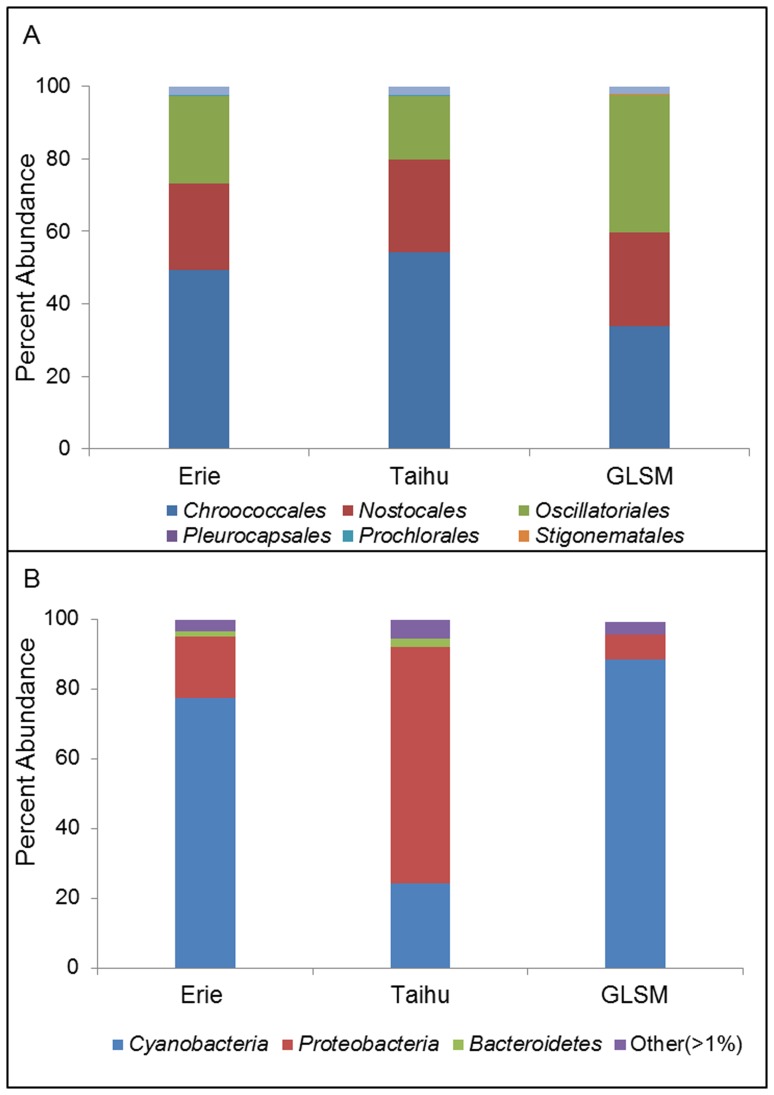
Identity of bloom-associated microbial community members. A) Percent abundance of orders belonging to the *Cyanobacteria* phylum. Abundance is a percentage of reads annotated as *Cyanobacteria* from each metagenome. B) Distribution of bacterial phyla in each metagenome. Abundance is a percentage of total bacterial reads in each metagenome. Phyla with less than 1% total abundance were grouped in the “Other” category.

**Table 2:** Percentage abundance of hits assigned to genera in the orders *Chroococcales* and *Oscillatoriales*.

	Erie	Taihu	GLSM
***Chroococcales***			
*Crocosphera-like*	9.4	9.2	6.7
*Cyanobium*	0.2	0.2	0.3
*Cyanothece*	71.9	72.9	69.5
*Microcystis*	1.6	2.4	0.8
*Synechococcus*	14.9	13.2	21.5
*Synechocystis*	0.6	0.6	0.3
*Unclassified*	1.3	1.5	0.9
***Oscillatoriales***			
*Arthrospira*	14.7	15.6	20.6
*Lyngbya*	31.1	21.4	27.6
*Microceleus*	18.0	26.6	13.2
*Planktothrix*	1.0	1.3	2.6
*Trichodesmium-like*	34.9	34.9	35.9

Sequences most closely identified as exclusively marine lineages (*Chrocosphera*, *Trichodesmium*) are denoted as “-like” and potentially are from related yet currently unclassified members of the community.

**Figure 4:**
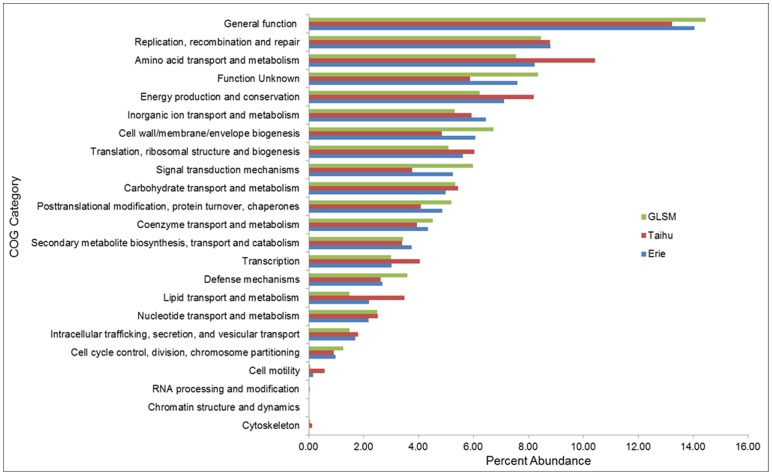
Abundance of metagenome reads assigned to each COG category. Metagenomes were annotated using the COG database. Reads were assigned to standard COG functional categories based on this annotation. Abundance is a percentage of total hits to the COG database.

## Results and Discussion

### The *Microcystis* Metagenome

It is widely known that not all members of the genus *Microcystis* have the genetic capability to produce microcystin – only 10–50% of cells in natural systems have these genes [17], [18]. It is thus likely that similar variability in content may be reflected in other genomic regions, a factor that is important to know when picking model organisms for lab study. Using SeqMan NGen® (DNAStar, Madison, WI), an initial attempt was made to generate whole genome assemblies of environmental *Microcystis* from each metagenome for genome-wide comparison. This effort was abandoned early however, as we realized there were issues with the potential recruitment of non-*Microcystis* reads to the sequenced lab isolate used as a scaffold. Instead, recruitment plots were generated in MUMmer 3.22 to identify potential genomic differences between bloom-associated *Microcystis* spp. and the sequenced lab isolate [40]. Sequences from each metagenome assigned a best hit identity of *Microcystis* were aligned to the *Microcystis aeruginosa* NIES 843 genome [41]. Recruitment of GLSM sequences was limited, with only 7% total coverage of the reference genome. Out of the possible 1327 individual *Microcystis* genes identified in the GLSM metagenome, 64% of genes had at least one repeat. (Figure 1A). The GLSM bloom has been subsequently characterized as a predominately *Planktothrix* bloom by microscopic and molecular analysis (Bullerjahn and McKay, unpublished), providing a foundation for our observation of limited recruitment to the NIES 843 genome. This characterization provides a valid explanation for the limited recruitment of *Microcystis* reads from the GLSM metagenome. Taihu sequences had greater recruitment to the reference genome compared to the GLSM data set, with 51% of the reference sequence covered (Figure 1B). 79% of the possible 4256 recruited sequences from Taihu were present at least twice in the metagenome data set. A robust recruitment, covering of 79% of the NIES 843 reference genome, resulted from alignment of Lake Erie sequences, with 92% of the 5539 *Microcystis* sequences recruited to the NIES 843 scaffold repeated at least once in the Erie metagenome (Figure 1C). This difference in recruitment between Erie and Taihu is likely due to differences in abundance of *Microcystis* spp. within our microbial samples. While the method of sample collection was the same across each bloom, collection took place at different points during bloom development at each site, which may have contributed to this difference in abundance of *Microcystis* spp.

**Figure 5:**
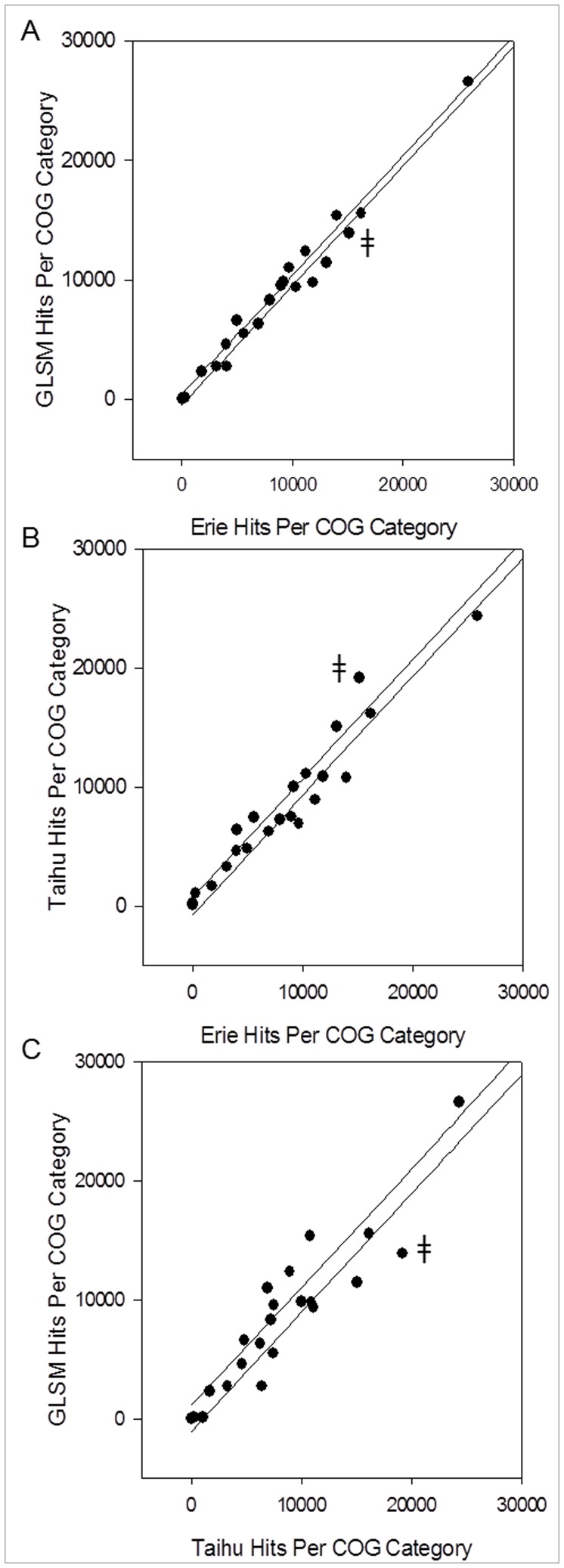
Comparison of number of hits to COG categories between bloom metagenomes. Normalized number of hits to each COG category from each metagenome plotted against the other. Data were normalized to the metagenome with the greatest number of hits to the COG database (Taihu). A) COG distributions of Erie and GLSM are compared. Data points largely fall within or close to the 95% confidence interval (CI) which is indicated by the black lines, indicating similarity. B) COG distributions of Erie and Taihu are compared. C) COG distributions of Taihu and GLSM are compared. Outliers in (B) and (C) are largely conserved, indicating closer conservation of COG distribution between the North American lakes to each other than Taihu. The largest outlier in the Taihu comparisons is denoted by the (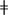) symbol in each plot. This outlier corresponds to the Amino Acid Metabolism and Transport COG category.

Six conserved metagenomic islands were identified in the Taihu and three in the Lake Erie recruitments (Figure 2A, 2B). All six regions initially identified as potential MIs are shown in Figure 2. Despite having only three regions that qualified as MIs, it is worth mentioning that the Lake Erie dataset does demonstrate reduced similarity and coverage in all of these regions. Metagenomic islands (MI’s) have been defined previously as areas within a genome that are underrepresented in metagenomic datasets. It is thought that MI’s represent regions of a genome that are unique between closely related strains, or components of the species pangenome [39]. For this analysis, these were identified as regions of 10 kb or larger that had relatively poor similarity and coverage (<25%) to the reference, consistent with similar analyses performed in marine environments [39]. For a detailed breakdown of the coverage of each MI, see Table S2. A number of transposases and hypothetical proteins were consistently identified as missing across all six islands: a complete list of genes within the six metagenomic islands is provided in Table S3. Within the second island, two genes involved in restriction modification systems failed to recruit. Interestingly, the *5S*, *16S*, and *23S* rRNA genes are located in the third island, although they recruited fully to the reference in both Taihu and Lake Erie recruitments. NIES 843 CRISPR-associated (Clustered Regularly Interspaced Short Palindromic Repeats) protein genes are located in the fifth metagenomic island, one of which failed to recruit from the Lake Erie metagenome. In the sixth and largest island (∼42 kb), a number of genes involved in cell surface recognition systems failed to recruit. These genes encoded for two cell surface antigen receptor proteins, a two component response regulator, and a sensor protein.

Taken together, the genes missing from each metagenomic island represent genes that may all be involved in phage recognition, consistent with metagenomic islands identified in marine and hypersaline environments [39], [42]. Although these gaps in recruitment may be an artifact of sequencing depth, this is unlikely, as there was robust recruitment to a vast majority to the NIES 843 genome by the Erie reads and islands were conserved between the datasets. The three MIs present in Taihu that did not meet the criteria in Erie may be examples of limited recruitment due to differences in population makeup between Erie and Taihu. Alternatively, these regions may differ from the reference, but are not yet divergent enough to qualify as MIs in Erie. The future establishment of a *Microcystis* spp. core genome (as more sequences become available) will be important in the verification of these differences. One explanation for the three islands conserved in both recruitments is that they represent regions of bloom-associated *Microcystis* genomes that are too divergent from the reference to recruit.

**Table 3:** The abundance of microbial nitrogen assimilation genes within metagenome.

	Erie						Taihu						GLSM					
	*nifD, H & K*	*ureA–G*	*nar/nir*	*glnA*	*rpoB*		*nifD, H & K*	*ureA–G*	*nar/nir*	*glnA*	*rpoB*		*nifD, H & K*	*ureA–G*	*nar/nir*	*glnA*	*rpoB*	
***Cyanobacteria***	210	84	0	17	399		41	26	0	8	80		201	126	0	48	371	
***Proteobacteria***	58	60	3	41	118		261	76	135	138	537		7	7	0	0	27	
***Actinobacteria***	–	–	–	–	0		–	–	–	2	1		–	–	–	3	3	
***Cytophaga***	–	–	–	6	1		–	–	–	–	–		–	–	–	–	–	
**Total Hits**	268	144	3	64	517		302	102	135	148	618		208	133	0	51	401	

Genes from families associated with nitrogen fixation (*nifD, nif H, nifK*), urea assimilation (*ureA–G*), nitrate reduction (*nar*, *nir*) and ammonium utilization (*glnA*) were identified and enumerated within each metagenome using the COG database and phylogenetically classified using RefSeq. Results represent the total number of hits to functional categories (COG database) within each library and are normalized for library size.

### Structure of the Co-occurring Community during *Microcystis* Blooms

Phylogenetic composition of each bloom sample was compared at multiple taxonomic levels. A relatively conserved phylogenetic structure was observed in the cyanobacterial populations for all of the lake samples (Figure 3A). The orders *Chroococcales*, *Nostocales*, and *Oscillatoriales* were most abundant in all three systems. The oscillatorians made up a slightly higher percentage of the total cyanobacterial population within GLSM (38%) relative to Erie (24%) or Taihu (17%). At the genus level, the most common best hit matches within the *Chroococcales* were the nitrogen-fixing *Cyanothece*, as well as *Synechococcus*, with *Microcystis* spp. making up less than three percent of the total population for each bloom. Despite their relatively low abundance, *Microcystis* spp. remain important as they were the dominant toxin producers in Lake Erie and Taihu (Table 2) [43], [44]. Genetic elements classifying to *Oscillatoria* were largely comprised of *Lyngbya* spp. and a group whose closest relatives are members of *Trichodesmium* at the genus level. Because *Trichodesmium* spp. are marine organisms, it is likely that a significant population of previously unclassified freshwater nitrogen fixers were present at high abundance in all three blooms. This finding is indicative of the need for more freshwater cyanobacterial genome sequences to more comprehensively study bloom events and other phenomena. As with members of *Chroococcales*, the dominant toxin producers within the oscillatorians (*Planktothrix*) are present in lower numbers than other genera (Table 2). At the genus level, phylogenetic resolution cannot be completely achieved, as current annotations reflect only what is currently available in the databases used for annotation, and are reported as the best hit. As databases continue to expand and more freshwater genomes become available, best hit annotations may change and give a more accurate representation of the communities. We include this information with the knowledge that as updates to databases and annotation algorithms improve, these assignments may change.

At the phylum level, microbial community composition varied across the different lakes (Figure 3B). *Cyanobacteria* were the most abundant members of the Erie and GLSM bloom samples (77% and 88%, respectively). The *Proteobacteria* were the second most abundant group in the North American lakes, followed by *Bacteroidetes* and several rare phyla (<1%). In Taihu, the *Proteobacteria* were the most abundant, despite higher measured levels of *chl* a (Table 1). Proteobacteria comprise 68% of the total microbial population, followed by *Cyanobacteria* at 25%, which is consistent with previous investigations of heterotrophic populations in the Taihu system [45], [46]. Further repetition of these datasets across multiple time points would provide more comprehensive insight into the bloom-associated community. As toxin-levels and the abundance of toxin-producers change throughout the course of a bloom, so may the associated bacterial community, as has been previously demonstrated in Taihu [47]. While possibly an artifact of sequencing coverage (a re-occurring theme in all metagenomics surveys), age of the bloom at the time of sample collection or of the sample collection techniques, these differences between Taihu and the North American lakes may be demonstrative of phylogenetic variability of *Microcystis*-associated bacteria between geographically distinct systems.

### Functional Potential of Co-occurring Heterotrophic Bacteria

Genes from each site were assigned to broad functional categories (Clusters of Orthologous Groups, COGs) and the relative abundance of hits to each category was compared across the three metagenomes (Figure 4). The number of genes within several categories was closely conserved across all sites. This includes genes involved in nutrient metabolism, coenzyme transport and metabolism, secondary metabolite metabolism and transport, as well as replication, recombination, and repair.

Despite this general conservation, there were multiple categories in which genes from the co-occurring microbial community in Taihu were over- or underrepresented compared to the North American lakes. The greatest differences in abundance were in the genes involved in amino acid metabolism and signal transduction mechanisms. The largest difference was ∼20% between Taihu and Erie/GLSM normalized COG distributions. Even with these differences, the functional potential of bloom community members appears relatively static between blooms, especially when compared to the striking phylogenetic variability between Taihu and the other systems.

To confirm the observed conservation of functional potential, the normalized number of hits to each COG category for each metagenome was plotted against the other (Figure 5). All points that fell within the calculated 95% confidence interval were assumed to be conserved in frequency of occurrence between the two blooms being compared. The only outlier in the comparison between the North American lakes was the category containing genes of unknown function (Figure 5A). As expected, the farthest outlier between Taihu and both Erie and GLSM was the Amino Acid Metabolism and Transport category (Figure 5B–C). Other conserved outliers included COGs for Signal Transduction Mechanisms, Energy Production & Conservation and Cell Wall/Membrane/Envelope Biogenesis. Taken with the original observational comparison of relative abundance of each COG category, these data suggest a conserved functional potential of the bloom-associated community, despite variable phylogenetic makeup. This provides support to the growing body of evidence suggesting that variable phylogenetic makeup may not be reflected in the function of the environmental microbial community members [1], [5].

Due to their potential role in the regulation of bloom formation in Taihu, we specifically took a deeper look at pathways associated with nitrogen cycling [48]. Functional genes involved in nitrogen assimilation were identified and annotated at the phylum level in each dataset to highlight the genomic contribution of bloom microbes to the incorporation of nitrogen in the community. Genes examined were those involved in nitrogen fixation (*nifD, nifH, nifK*), urea metabolism (*ureA–G*), nitrate and nitrite reduction (*nar* and *nir*), and glutamine synthesis (*glnA*). A stark contrast in the contribution of genes involved in nitrogen fixation and urea metabolism emerged between Taihu and the North American lakes (Table 3). *Cyanobacteria* contribute the majority of the genes involved in these processes in Erie (78% and 58%) and GLSM (96% and 95%). In contrast, *Proteobacteria* contribute an overwhelming majority of these functional genes (86% and 74%) within the Taihu bloom community. This highlights the potential for assimilation of nitrogen by different organisms in the bloom-associated community in Taihu relative to Erie and GLSM. It should be noted that amino acids may also serve as potential nitrogen sources, and the overrepresentation of those genes involved in general amino acid metabolism in Taihu may reflect an important role for heterotrophs in nitrogen metabolism in this system (Figure 4). The normalized abundance of genes involved in each type of nitrogen assimilation remains relatively constant, despite their taxonomic identity (Table 3). These data also highlight the somewhat divergent phylogenetic makeup of each community. The overrepresentation of nitrogen assimilation genes associated with *Proteobacteria* in Taihu is largely due to numerical dominance of these organisms. To demonstrate this, the commonly used housekeeping gene *rpoB* can be used as a crude proxy for organism abundance, and has been included to allow for normalization of nitrogen assimilation genes within phyla (Table 3) [49], [50]. While normalization changes the phylogenetic identity of the majority of the N assimilation genes, it again reiterates the possibility of phylogenetically distinct organisms having the potential to perform a similar functional role in bloom-associated microbial communities.

The data further illustrate an important caveat and area for future investigation: similar biological processes (*e.g.,* those discussed above) within different organisms are likely to result in different biochemical outcomes as these similar processes are independently coupled to unique subsequent process within members of the population. To this end our data need to be considered with a reminder of the importance of looking beyond individual pathways (or even parts of pathways) when trying to address biogeochemistry and the functional level.

This pattern in nitrogen assimilation gene distribution underscores the potential role of heterotrophic bacteria within toxic cyanobacterial bloom communities in some freshwater lakes. The role of heterotrophs in cyanobacterial metabolism has been previously observed in both marine and freshwater systems. Morris et al. (2008) [51] identified heterotrophic activity as the source of *Prochlorococcus* resistance to hydrogen peroxide in the oceans, while the important microcystin-producer *Planktothrix* has recently been found to successfully use the compound glyphosate, a herbicide known to be relatively resistant to biodegradation, as a nutrient source in the presence of “helper” heterotrophs [28]. Based on this metagenomic data, it appears that heterotrophs in Taihu may play an important role in nitrogen assimilation and transformation for the larger bloom microbial community.

### Conclusions

This work highlights the utility of metagenomics as a tool for exploration of microbial communities. Here, we provide microbial snapshots of three separate toxic cyanobacterial blooms. Despite being single samples, these metagenomes provide a unique snapshot of the microbial community associated with toxic cyanobacterial blooms. This initial characterization is an important foundation for further study of such communities. We have attempted to include metadata that complies with the Genomic Standards Consortium, with the intention that this initial characterization of three freshwater blooms can be reexamined for future statistical comparison as the repository of freshwater sequences builds [52]. While we focus largely on the bacterial community associated with bloom biomass, viral sequences were also present in all data sets. Notably, sequences of the *Microcystis* phage Ma-LMM01 were detected at all three lakes. This is especially worth noting due to the importance of phage in bloom dynamics and termination [53]. Other interesting findings include the presence of the *mlrC* gene in both Taihu and Erie. This gene is involved in microbial degradation of microcystin, and its presence warrants further inquiry into the presence of potential important microcystin degraders in these lakes [54].

In the current study we have used metagenomics to describe the phylogenetic makeup and functional potential of three geographically distinct cyanobacterial blooms at single time points during each bloom. This comparative approach has confirmed an increasing trend in microbial ecology: variable bacterial phylogenetic makeup is not mirrored in the relatively constant functional potential of the bacterial community in the environment [1]. This finding may reflect a need to amend current methods of describing microbial community dynamics, as the taxonomic/phylogenetic identity of community members may no longer be sufficient. Within our observations key functional genes, such as those involved in nitrogen assimilation, appear to be more informative than standard *16S* rDNA gene analysis and demonstrate that within 2 similar biological events (blooms in Lake Erie and Taihu) the analogous processes are likely carried out by different members of the community. With this approach, we were able to identify potentially divergent pathways of assimilated nitrogen through the microbial communities of three different blooms. The genomic contribution of heterotrophic bacteria to nitrogen assimilation in Taihu represents a potentially critical contribution of heterotrophic bacteria in driving toxic freshwater blooms.

## Supporting Information

Table S1 **Information regarding the sequence data sets used in this study.**
(DOCX)

Table S2 **Coverage of** ***Microcystis aeruginosa*** **NIES 843 metagenomic islands (MIs) identified in the Erie and Taihu datasets.**
(DOCX)

Table S3 **All genes present in each metagenomic island (MI) of M. aeruginosa NIES 843. Genes with and without coverage are listed.**
(DOCX)
