# Supplementary material for: Comparative Metagenomics of Toxic Freshwater Cyanobacteria Bloom Communities on Two Continents
Source: PLoS One. 2012 Aug 29;7(8):e44002. doi: 10.1371/journal.pone.0044002 (PMC3430607; doi:10.1371/journal.pone.0044002)
Supplement: Table S1 — Information regarding the sequence data sets used in this study. (DOCX) [file pone.0044002.s001.docx]

|  | Lake Erie |  | Taihu |  | GLSM |  |
| --- | --- | --- | --- | --- | --- | --- |
|  | Pre-QC | Post-QC | Pre-QC | Post-QC | Pre-QC | Post-QC |
| Total Reads | 504,223 | 354,384 | 637,724 | 401,996 | 219,716 | 140,179 |
| Total bp | 204,427,100 | 153,986,130 | 240,405,900 | 163,139,593 | 88,229,075 | 60,656,381 |
| Mean sequence length | 405 ± 133 | 434 ± 104 | 376 ± 127 | 405 ± 105 | 401 ± 141 | 432 ± 113 |
| GC Content | 46 ± 10% | 46 ± 10% | 51 ± 13% | 51 ± 13% | 41 ± 8% | 41 ± 8% |
