# Supplementary material for: Comparative Metagenomics of Toxic Freshwater Cyanobacteria Bloom Communities on Two Continents
Source: PLoS One. 2012 Aug 29;7(8):e44002. doi: 10.1371/journal.pone.0044002 (PMC3430607; doi:10.1371/journal.pone.0044002)
Supplement: Table S2 — Coverage of Microcystis aeruginosa NIES 843 metagenomic islands (MIs) identified in the Erie and Taihu datasets. (DOCX) [file pone.0044002.s002.docx]

|  | | **MI 1** | **MI 2** | **MI 3** | **MI 4** | **MI 5** | **MI 6** |
| --- | --- | --- | --- | --- | --- | --- | --- |
|  | |  |  |  |  |  |  |
| **Coordinates** |  | 120122..130126 | 421251..460015 | 1874286..1899881 | 2234993..2246392 | 2813246..2830250 | 3725469..3768893 |
|  |  |  |  |  |  |  |  |
| **% with no coverage** | *Erie* | 75.8% | 54.8% | 66.9% | 91.8% | 80.6% | 57.0% |
|  |  |  |  |  |  |  |  |
|  | *Taihu* | 88.7% | 90.7% | 76.7% | 95.8% | 85.2% | 80.6% |
|  |  |  |  |  |  |  |  |
| **Average % similarity with 843 reference** | *Erie* | 21.9% | 37.3% | 29.0% | 7.4% | 16.9% | 38.8% |
|  |  |  |  |  |  |  |  |
|  | *Taihu* | 10.4% | 8.4% | 19.8% | 3.6% | 12.8% | 16.6% |
