# Supplementary material for: Comparative Metagenomics of Toxic Freshwater Cyanobacteria Bloom Communities on Two Continents
Source: PLoS One. 2012 Aug 29;7(8):e44002. doi: 10.1371/journal.pone.0044002 (PMC3430607; doi:10.1371/journal.pone.0044002)
Supplement: Table S3 — All genes present in each metagenomic island (MI) of M. aeruginosa NIES 843. Genes with and without coverage are listed. (DOCX) [file pone.0044002.s003.docx]

| Island | Coordinates | Gene | Description |
| --- | --- | --- | --- |
|  |  |  |  |
| MI-1 | 120122..120571 | MAE_01450 | YP_001655159.1: transposase |
|  | 121213..121416 | MAE_01470 | YP_001655161.1: hypothetical protein |
|  | 121415..121675 | MAE_01480 | YP_001655162.1:transposase |
|  | 122392..123021 | MAE_01500 | YP_001655164.1:hypothetical protein |
|  | 123219..123779 | MAE_01510 | YP_001655165.1: hypothetical protein |
|  | 123822..124133 | MAE_01520 | YP_001655166.1: hypothetical protein |
|  | 124309..125967 | MAE_01530 | YP_001655167.1: transposase |
|  | 126308..126502 | MAE_01540 | YP_001655168.1: hypothetical protein |
|  | 126676..16984 | MAE_01550 | YP_001655169.1: hypothetical protein |
|  | 127447..128661 | MAE_01560 | YP_001655170.1: transposase |
|  | 129011..129658 | MAE_01570 | YP_001655171.1: hypothetical protein |
|  | 129735..129971 | MAE_01580 | YP_001655172.1: hypothetical protein |
|  | 129968..130126 | MAE_01590 | YP_001655173.1: hypothetical protein |
|  |  |  |  |
| MI-2 | 421251..421613 | MAE_04740 | YP_001655488.1: hypothetical protein |
|  | 421618..421785 | MAE_04750 | YP_001655489.1: hypothetical protein |
|  | 422089..422292 | MAE_04760 | YP_001655490.1: hypothetical protein |
|  | 422466..422627 | MAE_04770 | YP_001655491.1: hypothetical protein |
|  | 422976..423179 | MAE_04780 | YP_001655492.1: hypothetical protein |
|  | 423515..425173 | MAE_04790 | YP_001655493.1: transposase |
|  | 427533..428129 | MAE_04800 | YP_001655494.1: tranposase |
|  | 428328..428603 | MAE_04810 | YP_001655495.1: tranposase |
|  | 428570..429838 | MAE_04820 | YP_001655496.1: tranposase |
|  | 430411..431406 | MAE_04830 | YP_001655497.1: tranposase |
|  | 431565..432356 | MAE_04840 | YP_001655498.1: transposase |
|  | 433373..434521 | MAE_04850 | YP_001655499.1: transposase |
|  | 434292..434907 | MAE_04860 | YP_001655500.1: hypothetical protein |
|  | 435199..435573 | MAE_04870 | YP_001655501.1: hypothetical protein |
|  | 435914..436132 | MAE_04880 | YP_001655502.1:hypothetical protein |
|  | 436515..437135 | MAE_04890 | YP_001655503.1: hypothetical protein |
|  | 437381..437710 | MAE_04900 | YP_001655504.1: hypothetical protein |
|  | 437821..437982 | MAE_04910 | YP_001655505.1: hypothetical protein |
|  | 437985..438341 | MAE_04920 | YP_001655506.1:hypothetical protein |
|  | 438410..438904 | MAE_04930 | YP_001655507.1: SixA type phosphohistidine phosphatase |
|  | 438957..440177 | MAE_04940 | YP_001655508.1: hypothetical protein |
|  | 440645..442303 | MAE_04950 | YP_001655509.1: transposase |
|  | 442341..442697 | MAE_04960 | YP_001655510.1: hypothetical protein |
|  | 443212..443979 | MAE_04970 | YP_001655511.1: hypothetical protein |
|  | 444081..445739 | MAE_04980 | YP_001655512.1: transposase |
|  | 446868..447023 | MAE_04990 | YP_001655513.1: hypothetical protein |
|  | 447147..448025 | MAE_05000 | YP_001655514.1: type I restriction-modification system |
|  | 448235..449293 | MAE_05010 | YP_001655515.1: type I restriction-modification system |
|  | 449329..449748 | MAE_05020 | YP_001655516.1: hypothetical protein |
|  | 449857..450027 | MAE_05030 | YP_001655517.1: hypothetical protein |
|  | 450156..450509 | MAE_05040 | YP_001655518.1: putative restriction modification system |
|  | 451244..451651 | MAE_05050 | YP_001655519.1: putative restriction modification system |
|  | 451737..452939 | MAE_05060 | YP_001655520.1: type I site-specific deoxyribonuclease |
|  | 453090..454358 | MAE_05070 | YP_001655521.1: transposase |
|  | 454486..454752 | MAE_05080 | YP_001655522.1: hypothetical protein |
|  | 454809..455483 | MAE_05090 | YP_001655523.1: tranposase |
|  | 455519..456031 | MAE_05100 | YP_001655524.1: transposase |
|  | 456171..456329 | MAE_05110 | YP_001655525.1: hypothetical protein |
|  | 456375..457418 | MAE_05120 | YP_001655526.1: trasposase |
|  | 457485..457640 | MAE_05130 | YP_001655527.1: type I site-specific deoxyribonuclease |
|  | 457842..459326 | MAE_05140 | YP_001655528.1: transposase |
|  | 459399..459551 | MAE_05150 | YP_001655529.1: hypothetical protein |
|  | 459602..460015 | MAE_05160 | YP_001655530.1: hypothetical protein |
|  |  |  |  |
| MI-3 | 1874286..1875272 | MAE_21010 | YP_001657115.1: transposase |
|  | 1876106..1876348 | MAE_21020 | YP_001657116.1: hypothetical protein |
|  | 1876960..1878228 | MAE_21030 | YP_001657117.1: transposase |
|  | 1878296..1878805 | MAE_21040 | YP_001657118.1: transposase |
|  | 1878992..1879162 | MAE_21050 | YP_001657119.1: hypothetical protein |
|  | 1879166..1879513 | MAE_21060 | YP_001657120.1: transposase |
|  | 1879528..1879953 | MAE_21070 | YP_001657121.1: hypothetical protein |
|  | 1880244..1881512 | MAE_21080 | YP_001657122.1: transposase |
|  | 1881671..1883329 | MAE_21090 | YP_001657123.1: transposase |
|  | 1883916..1884215 | MAE_21100 | YP_001657124.1: hypothetical protein |
|  | 1884348..1885373 | MAE_21110 | YP_001657125.1: hypothetical protein |
|  | 1885475..1885699 | MAE_21120 | YP_001657126.1: hypothetical protein |
|  | 1885814..1887290 | rrn16S_1 | rRNA: 16S ribosomal RNA |
|  | 1887428..1887504 | MAE_t009 | tRNA:Ile |
|  | 1887656..1890533 | rrn23S_1 | rRNA: 23S ribosomal RNA |
|  | 1890592..1890709 | rrn5S_1 | rRNA: 5S ribosomal RNA |
|  | 1890758..1891048 | MAE_21130 | YP_001657127.1: hypothetical protein |
|  | 1891573..1892130 | MAE_21140 | YP_001657128.1: AMP-dependent synthetase and ligase |
|  | 1892134..1892340 | MAE_21150 | YP_001657129.1: hypothetical protein |
|  | 1892333..1893949 | MAE_21160 | YP_001657130.1: AMP-dependent synthetase and ligase |
|  | 1893968..1895290 | MAE_21170 | YP_001657131.1: class I/II aminotransferase |
|  | 1895388..1896506 | MAE_21180 | YP_001657132.1: glycine amidinotransferase |
|  | 1896517..1897185 | MAE_21190 | YP_001657133.1: hypothetical protein |
|  | 1897182..1898087 | MAE_21200 | YP_001657134.1: short chain dehydrogenase |
|  | 1898301..1898501 | MAE_21210 | YP_001657135.1: hypothetical protein |
|  | 1898655..1899881 | MAE_21220 | YP_001657136.1: ABC-transporter DevB family protein |
|  |  |  |  |
| MI-4 | 2234993..2235154 | MAE_24710 | YP_001657485.1: hypothetical protein |
|  | 2235494..2235691 | MAE_24720 | YP_001657486.1: hypothetical protein |
|  | 2235852..2236169 | MAE_24730 | YP_001657487.1: hypothetical protein |
|  | 2236166..2236513 | MAE_24740 | YP_001657488.1: hypothetical protein |
|  | 2236609..2237040 | MAE_24750 | YP_001657489.1: hypothetical protein |
|  | 2237041..2237427 | MAE_24760 | YP_001657490.1: hypothetical protein |
|  | 2237807..2237959 | MAE_24770 | YP_001657491.1: hypothetical protein |
|  | 2238032..2239516 | MAE_24780 | YP_001657492.1: transposase |
|  | 2239745..4420059 | MAE_24790 | YP_001657493.1: hypothetical protein |
|  | 2240067..2241335 | MAE_24800 | YP_001657494.1: transposase |
|  | 2241507..2243948 | MAE_24810 | YP_001657495.1: hypothetical protein |
|  | 2243948..2246392 | MAE_24820 | YP_001657496.1: hypothetical protein |
|  |  |  |  |
| MI-5 | 2813246..2814250 | cas1 | YP_001658100.1: CRISPR-associated Cas1 family protein |
|  | 2814263..2814535 | cas2 | YP_001658101.1: CRISPR-associated Cas2 family protein |
|  | 2814598..2814795 | MAE_30880 | YP_001658102.1: hypothetical protein |
|  | 2816187..2816363 | MAE_30890 | YP_001658103.1: hypothetical protein |
|  | 2816380..2816556 | MAE_30900 | YP_001658104.1: hypothetical protein |
|  | 2816668..2816826 | MAE_30910 | YP_001658105.1: hypothetical protein |
|  | 2817526..2817696 | MAE_30920 | YP_001658106.1: hypothetical protein |
|  | 2818072..2818236 | MAE_30930 | YP_001658107.1: hypothetical protein |
|  | 2819008..2819244 | MAE_30940 | YP_001658108.1: hypothetical protein |
|  | 2820299..2820469 | MAE_30950 | YP_001658109.1: hypothetical protein |
|  | 2821343..2821627 | MAE_30960 | YP_001658110.1: hypothetical protein |
|  | 2821850..2822017 | MAE_30970 | YP_001658111.1: hypothetical protein |
|  | 2822231..2822392 | MAE_30980 | YP_001658112.1: hypothetical protein |
|  | 2822939..2823109 | MAE_30990 | YP_001658113.1: hypothetical protein |
|  | 2823272..2823433 | MAE_31000 | YP_001658114.1: hypothetical protein |
|  | 2823480..2823662 | MAE_31010 | YP_001658115.1: hypothetical protein |
|  | 2823748..2824011 | MAE_31020 | YP_001658116.1: hypothetical protein |
|  | 2824060..2824242 | MAE_31030 | YP_001658117.1: hypothetical protein |
|  | 2084735..2825247 | MAE_31040 | YP_001658118.1: transposase |
|  | 2825283..2825957 | MAE_31050 | YP_001658119.1: transposase |
|  | 2826014..2826190 | MAE_31060 | YP_001658120.1: hypothetical protein |
|  | 2826399..2826743 | MAE_31070 | YP_001658121.1: hypothetical protein |
|  | 2826940..2827149 | MAE_31080 | YP_001658122.1: hypothetical protein |
|  | 2828121..2828393 | MAE_31090 | YP_001658123.1: hypothetical protein |
|  | 2828631..2828801 | MAE_31100 | YP_001658124.1: hypothetical protein |
|  | 2829465..2829752 | MAE_31110 | YP_001658125.1: hypothetical protein |
|  | 2829903..2830250 | MAE_31120 | YP_001658126.1: hypothetical protein |
|  |  |  |  |
| MI-6 | 3725469..3726938 | MAE_40650 | YP_001659079.1: hypothetical protein |
|  | 3726941..3727795 | MAE_40660 | YP_001659080.1: hypothetical protein |
|  | 3727825..3729195 | MAE_40670 | YP_001659081.1: von Willebrand factor type A |
|  | 3729113..3730219 | MAE_40680 | YP_001659082.1: hypothetical protein |
|  | 3730231..3732540 | MAE_40690 | YP_001659083.1: hypothetical protein |
|  | 3732621..3735221 | MAE_40700 | YP_001659084.1: hypothetical protein |
|  | 3735226..3736140 | MAE_40710 | YP_001659085.1: hypothetical protein |
|  | 3736127..3736972 | MAE_40720 | YP_001659086.1: hypothetical protein |
|  | 3736992..3737765 | MAE_40730 | YP_001659087.1: hypothetical protein |
|  | 3738093..3738800 | MAE_40740 | YP_001659088.1: hypothetical protein |
|  | 3738822..3739733 | MAE_40750 | YP_001659089.1: hypothetical protein |
|  | 3740070..3740693 | MAE_40760 | YP_001659090.1: hypothetical protein |
|  | 3740745..3741206 | MAE_40770 | YP_001659091.1: hypothetical protein |
|  | 3741197..3741673 | MAE_40780 | YP_001659092.1: hypothetical protein |
|  | 3741679..3742743 | MAE_40790 | YP_001659093.1: hypothetical protein |
|  | 3743368..3743721 | MAE_40800 | YP_001659094.1: hypothetical protein |
|  | 3744056..3746173 | MAE_40810 | YP_001659095.1: serine/threonine protein kinase |
|  | 3746285..3748108 | MAE_40820 | YP_001659096.1: protein serine/threonine phosphatase |
|  | 3748173..3748736 | MAE_40830 | YP_001659097.1: FHA domain-containing protein |
|  | 3748825..3750207 | MAE_40840 | YP_001659098.1: von Willebrand factor type A |
|  | 3750231..3750623 | MAE_40850 | YP_001659099.1: hypothetical protein |
|  | 3750643..3752817 | MAE_40860 | YP_001659100.1: von Willebrand factor type A |
|  | 3752923..3755109 | MAE_40870 | YP_001659101.1: hypothetical protein |
|  | 3755157..3755381 | MAE_40880 | YP_001659102.1: hypothetical protein |
|  | 3755965..3756459 | MAE_40890 | YP_001659103.1: hypothetical protein |
|  | 3756465..3757922 | MAE_40900 | YP_001659104.1: HlyD family secretion protein |
|  | 3758217..3758819 | MAE_40910 | YP_001659105.1: hypothetical protein |
|  | 3758903..3759883 | pyrB | YP_001659106.1: aspartate carbamoyltransferase catalytic subunit |
|  | 3759907..3760188 | MAE_40930 | YP_001659107.1: hypothetical protein |
|  | 3760379..3760555 | MAE_40940 | YP_001659108.1: hypothetical protein |
|  | 3760670..3761503 | MAE_40950 | YP_001659109.1: surface antigen D15 |
|  | 3761957..3762454 | MAE_40960 | YP_001659110.1: surface antigen variable number |
|  | 3762465..3763106 | MAE_40970 | YP_001659111.1: two-component response regulator |
|  | 3763339..3763632 | MAE_40980 | YP_001659112.1: hypothetical protein |
|  | 3763974..3764159 | MAE_40990 | YP_001659113.1: hypothetical protein |
|  | 3764204..3764371 | MAE_41000 | YP_001659114.1: hypothetical protein |
|  | 3764646..3765110 | MAE_41010 | YP_001659115.1: hemolysin-type calcium-binding protein |
|  | 3765371..3767299 | MAE_41020 | YP_001659116.1: sensor protein |
|  | 3767424..3768173 | MAE_41030 | YP_00165117.1: hypothetical protein |
|  | 3768246..3768893 | MAE_41040 | YP_00165118.1: hypothetical protein |
